# What Happened to People with Non-Communicable Diseases during COVID-19: Implications of H-EDRM Policies

**DOI:** 10.3390/ijerph17155588

**Published:** 2020-08-03

**Authors:** Emily Ying Yang Chan, Jean Hee Kim, Eugene Siu Kai Lo, Zhe Huang, Heidi Hung, Kevin Kei Ching Hung, Eliza Lai Yi Wong, Eric Kam Pui Lee, Martin Chi Sang Wong, Samuel Yeung Shan Wong

**Affiliations:** 1 Collaborating Centre for Oxford University and CUHK for Disaster and Medical Humanitarian Response (CCOUC), The Chinese University of Hong Kong, Hong Kong, China; Euglsk@cuhk.edu.hk (E.S.K.L.); huangzhe@cuhk.edu.hk (Z.H.); kevin.hung@cuhk.edu.hk (K.K.C.H.); 2 Nuffield Department of Medicine, University of Oxford, Oxford OX37BN, UK; 3 JC School of Public Health and Primary Care, The Chinese University of Hong Kong, Hong Kong, China; JHKim@cuhk.edu.hk (J.H.K.); heidihung@link.cuhk.edu.hk (H.H.); lywong@cuhk.edu.hk (E.L.Y.W.); lkp032@cuhk.edu.hk (E.K.P.L.); wong_martin@cuhk.edu.hk (M.C.S.W.); yeungshanwong@cuhk.edu.hk (S.Y.S.W.); 4 Accident & Emergency Medicine Academic Unit, The Chinese University of Hong Kong, Prince of Wales Hospital, Hong Kong, China

**Keywords:** Health-EDRM, non-communicable disease, COVID-19, self-care, NCD management, home care, early phase of pandemic

## Abstract

People with existing non-communicable diseases (NCDs) are particularly vulnerable to health risks brought upon by emergencies and disasters, yet limited research has been conducted on disease management and the implications of Health-EDRM policies that address health vulnerabilities of people with NCDs during the COVID-19 pandemic. This paper reports the baseline findings of an anonymous, random, population-based, 6-month cohort study that aimed to examine the experiences of people with NCDs and their relevant self-care patterns during the COVID-19 pandemic. A total of 765 telephone interviews were completed from 22nd March to 1st April 2020 in Hong Kong, China. The dataset was representative of the population, with 18.4% of subjects reporting at least one NCD. Results showed that low household income and residence in government-subsidized housing were significant predictors for the subjects who experienced difficulty in managing during first 2 months of the pandemic (11% of the NCD patients). Of those on long-term NCD medication, 10% reported having less than one week’s supply of medication. Targeted services for vulnerable groups during a pandemic should be explored to support NCD self-care.

## 1. Introduction

People with existing non-communicable diseases (NCDs) are particularly vulnerable to health risks brought upon by emergencies and disasters [1]. People who suffer from chronic diseases, such as cardiovascular disease, chronic lung disease, and diabetes, are more vulnerable to disruption and stress induced by disasters. A significant proportion of mortality in post-disaster phases results from the failure of health care services to cater to the needs of patients with chronic diseases [2].

The Health emergency and disaster risk management (Health-EDRM) framework emphasizes prevention and risk mitigation through hazard and vulnerability reduction, disaster preparedness, and response and recovery measures [3]. As the presence of NCDs are reported to be associated with worse outcomes of the COVID-19 disease, [4,5,6] strengthening NCD self-care and disease management during the pandemic could mitigate the health harm caused by COVID-19. In addition to the maintenance of healthy behaviours (e.g., regular exercise, personal hygiene), NCD patients should continue their regular medication and are recommended to stockpile at least one-month’s supply of medication during the pandemic [7]. Ironically, some of the infection control measures, such as lockdowns, and the reallocation of healthcare resources to handle COVID-19 cases, have posed challenges for maintaining care among NCD patients.

The impact of COVID-19 on NCD management has caused global concerns, and the European WHO Regional Office has begun devising recommended actions for people with NCDs during this pandemic [8]. However, research on the status and disease management of NCD patients in the context of COVID-19 remains very limited. This study examines the situation of people with NCDs, their disease management difficulties, and household supply of medication during the early phase of the pandemic. The most vulnerable NCD patient subgroups were identified and discussed.

## 2. Materials and Methods

This is an anonymous, random, population-based, 6-month cohort study. This report highlights findings of the baseline data collection (22nd March to 1st April 2020). Participants were recruited through computerized random digit dialing (RDD). Stratified sampling was used to ensure that the dataset was representative of the Hong Kong general population in terms of age group, gender, and district of residence. Details of the methodology were reported in our previous study [9], which investigated the perception, attitude and preparation for the COVID-19 epidemic among the Hong Kong population. Data were collected in Hong Kong, a southern metropolis in China, and the health services delivery was in an urban setting.

The study population included those aged 18 years or above and residing in Hong Kong. Socio-demographic data (age, gender, household income, employment status, housing type) and details of NCD patients’ disease management situation (presence and types of chronic condition(s), healthcare services utilization, routine care requirements) were collected by a standardized questionnaire. Participants were also asked about their past medical history and whether their family members had chronic condition(s). An NCD was defined as a self-reported, existing, chronic condition through the questions “Do you suffer from any form of chronic disease?” and “Which type of the chronic disease(s) are you diagnosed”. Households reporting at least one member with an NCD were asked if they had at least one week’s supply of NCD medications at home during the COVID-19 pandemic. In addition, they were further asked whether the COVID-19 pandemic had caused difficulty to their usual NCD care and the nature of these difficulties [9].

Differences between participants with and without perceived difficulty in their usual NCD care during the COVID-19 pandemic were examined by Chi-square tests and Fisher’s exact tests (α = 0.05). Respondents gave verbal informed consent and the study was approved by the Survey and Behavioral Research Ethics Committee at The Chinese University of Hong Kong (SBRE-19-498).

## 3. Results

Our telephone survey reached 765 households, and the final response rate was 44.0% (765/1738). Our sample was comparable to the Hong Kong general population [9]. Of all the households interviewed, 31.5% reported the presence of at least one person in the household diagnosed with an NCD, and among them, 9.1% reported not having at least one week’s supply of NCD medications at the time of phone interview.

Of all the participants, 18.4% (*n* = 141) reported having at least one type of NCD, and approximately 5% (or 27% of these patients) reported more than one type of NCD. Approximately 44.7% of these NCD patients were aged 65 or above. The most commonly reported NCDs were hypertension (48.6%), diabetes (22.1%), cardiovascular diseases (16.4%), and hyperlipidemia (10.0%). Of NCD patients, nearly four-fifths (*n* = 110) had required medication(s) for their condition.

**Figure 1:**
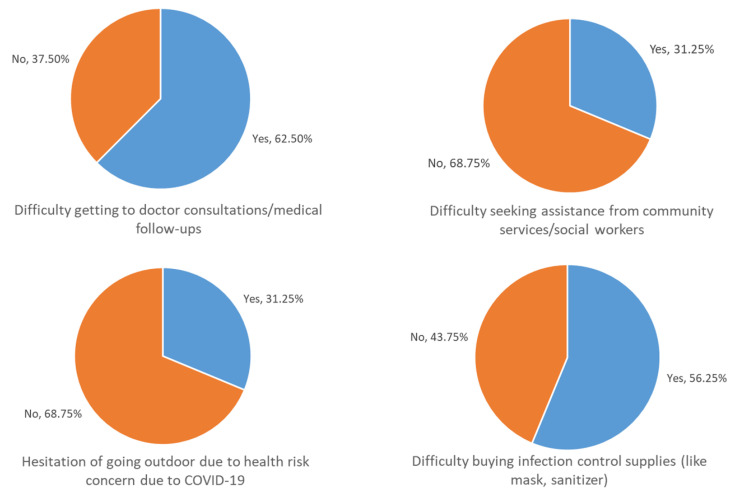
Difficulties reported by study samples for non-communicable diseases (NCD) management during COVID-19 pandemic (*n* = 16).

Around 11% of participants with NCDs reported difficulty in their routine NCD care, with the most common reasons being difficulty in getting to medical consultations/follow-up visits during the pandemic (62.5%) and difficulty in purchasing supplies, such as face masks and hand sanitizers, during this period (56.3%) (Figure 1). Among participants who reported difficulties in NCD management, those with lower income and those living in government-subsidized housing were more likely to perceive difficulties in NCD management (Table 1), while no statistically significant differences were noted for other demographic variables. The results also revealed no statistically significant difference between participants with different types of NCD or between patients with one NCD versus multiple NCDs.

## 4. Discussion

In our study, we found that around one-fifth of the Hong Kong population reported to have NCDs. Among those NCD patients, lower household income and residing in government-subsidized housing were found to be significantly associated with difficulty in NCD management during the first two months of the pandemic. In addition, households with NCD patients were reasonably well-prepared in terms of medication stockpiling during the COVID pandemic in Hong Kong, with over 90% possessing at least a week’s supply of drugs. Moreover, all public outpatient clinics were open during COVID-19 with enhanced infection control measures, and allowed relatives/friends to obtain drugs for NCDs on the patients’ behalf. Nonetheless, nearly one in ten NCD patients were insufficiently prepared with their medication supply.

Previous studies indicated that social distancing and quarantine could result in poor management of NCD behavioral risk factors, including various unhealthy lifestyle habits [10]. In particular, reduced social interaction, uncertainty in economic situations, and changes in the activities of daily living could further worsen disease management among NCD patients [8]. While only about 11% of the NCD patients in this study reported perceived difficulties in managing their NCD during the pandemic, the results indicate the pandemic disrupted access to NCD clinical care, possibly due to services/traffic interruption and difficulties arising from rescheduling of routine check-ups. NCD patients of lower income and those living in government-subsidized housing were significantly more likely to perceive difficulty in NCD management during the pandemic, indicating that material resources may be major barriers to care. A possible reason may be that since the willingness to wear face masks to prevent infection transmission in Hong Kong is high (e.g., around 90% of Hong Kong residents wore mask during the A/H5N1 avian influenza period in 2007 and A/H1N1 influenza period in 2009 [11]), it is not surprising that the most commonly reported difficulties for NCD care are getting to medical consultations/follow-up visits and purchasing medical supplies, given the soaring price of face masks in the first few months of the pandemic [12]. Thus, NCD patients should thereby receive more targeted services to facilitate their NCD self-care during a pandemic. Further studies, in particular on telemedicine, can investigate interventions to minimize such NCD management interruptions [13].

Health-EDRM concerns the analysis and management of health risks through reduction in hazard, exposure and vulnerability in every phase of the disaster management cycle [14]. Resilience-building is a key concept for minimizing the health risks of older people and chronic disease patients, and could be built through empowerment initiatives to improve their health outcomes. Self-care by the population concerned should also be promoted. For chronic disease patients, they should have adequate knowledge on how to use their medication (e.g., type of insulin used, insulin self-injection kit with instructions). For people with multiple drug prescriptions, it would be important for them to identify the critical, life-maintaining ones, and the key contraindications of their regular medications. In the event that health facilities and medical supplies are interrupted, it is important for chronic disease patients to stockpile, preferably, a 10–14 days’ supply of medications [15]. Moreover, extensive effort is required to promote emergency preparedness among chronic disease patients, as a systematic review published in 2014 found that a considerable number of chronic disease patients lost their medication and medical aids during evacuation. Many did not bring prescriptions with them when evacuated, which made it difficult to fill in prescriptions, and that medication and prescription loss posed a significant burden on the medical relief teams [16]. Community partnership is crucial, and health care workers who are involved in disaster response and relief should be sensitive in choosing the most appropriate NCD health interventions (e.g., adverse drug interactions, unsuitable diets for people with diabetes [17]) to support patients during extreme events.

There were some limitations of this study. For the question of NCD medication stockpiling, since the interview could be answered by the patient’s family members, the accuracy could be undermined by recall bias. In addition, the sample size of the chronic disease patients was very small and did not permit multivariable analysis. Although the results provided some initial insight into NCD healthcare needs and service gaps in a region that was affected by the early phase of the COVID-19 pandemic, the representativeness of the NCD subsample to the general population of NCD patients is unknown. The results should therefore be taken with caution, and future studies need to capture a larger, representative sample of NCDs patients for examination. In order to capture a representative sample of NCD patients with high quality data, a future study should be conducted by randomly sampling NCD patients from patient lists (which provides investigators with documentation of the clinical diagnoses and prescribed medications). These patients can be followed periodically during the epidemic in order to track changes in their healthcare needs and service gaps in the early and later phases. The time effect of self-reported disease management patterns will be examined in the second phase of data collection. Future studies should also examine the impacts of large-scale pandemics and public health emergencies on long-term NCD management.

## 5. Conclusions

This study examines the disease management difficulties faced by NCD patients during the early phase (first 2 months) of the COVID-19 pandemic and identified the most vulnerable NCD patient subgroups in an urban context. Study findings indicated low household income and residence in government-subsidized housing were found to be significant predictors among the 11% who reported difficultly in managing during first 2 months of the pandemic. Of those on long-term NCD medication, 10% reported having less than one week’s supply of medication. Targeted services for vulnerable groups during a pandemic should be explored to facilitate resilience-building in Health-EDRM and to enable better self-care for people with NCDs.

## Figures and Tables

**Table 1:** Perceived difficulties for NCD management by sociodemographic factors during the COVID-19 pandemic.

	Perceived No Difficulty (*n* = 125)	Perceived Difficulty (*n* = 16)	*p*-Value
**Gender**			0.394
Male	48.8%	37.5%	
Female	51.2%	62.5%	
**Age ^a^**			0.307
18–24	3.2%	0.0%	
25–44	7.2%	18.8%	
45–64	45.6%	31.3%	
65 or above	44.0%	50.0%	
**Education attainment ^a^**			0.087
Primary level or below	14.5%	18.8%	
Secondary level	54.0%	75.0%	
Tertiary level	31.5%	6.3%	
**Living alone ^a^**			0.224
Not living alone	85.6%	100.0%	
Living alone	14.4%	0.0%	
**Employment group ^a^**			0.289
White collar	24.2%	6.3%	
Blue collar	14.5%	31.3%	
Housewives	20.2%	25.0%	
Students	1.6%	0.0%	
Unemployment or retired	39.5%	37.5%	
**Housing type ^a^**			0.012 *
Public housing	32.0%	18.8%	
Government subsidized housing	10.4%	31.3%	
Private housing	57.6%	43.8%	
Others	0.0%	6.3%	
**Monthly h ousehold income (HK$) ^a,b^**			0.018 *
< 8000	22.8%	12.5%	
8000–19999	25.4%	62.5%	
20000–39999	20.2%	18.8%	
40000 or more	31.6%	6.3%	

^a^ Fisher’s exact test. ^b^ USD = 7.8 HKD. * *p* < 0.05.
